# Parasites and vector-borne diseases disseminated by rehomed dogs

**DOI:** 10.1186/s13071-020-04407-5

**Published:** 2020-11-10

**Authors:** Ian Wright, Frans Jongejan, Mary Marcondes, Andrew Peregrine, Gad Baneth, Patrick Bourdeau, Dwight D. Bowman, Edward B. Breitschwerdt, Gioia Capelli, Luís Cardoso, Filipe Dantas-Torres, Michael J. Day, Gerhard Dobler, Lluis Ferrer, Luigi Gradoni, Peter Irwin, Volkhard A. J. Kempf, Barbara Kohn, Friederike Krämer, Michael Lappin, Maxime Madder, Ricardo G. Maggi, Carla Maia, Guadalupe Miró, Torsten Naucke, Gaetano Oliva, Domenico Otranto, Maria Grazia Pennisi, Barend L. Penzhorn, Martin Pfeffer, Xavier Roura, Angel Sainz, SungShik Shin, Laia Solano-Gallego, Reinhard K. Straubinger, Séverine Tasker, Rebecca Traub, Susan Little

**Affiliations:** 1 The Mount Veterinary Practice, Fleetwood, UK; 2 Department of Veterinary Tropical Diseases, University of Pretoria, Onderstepoort, South Africa; 3 School of Veterinary Medicine, São Paulo State University, São Paulo, Brazil; 4 Department of Pathobiology, University of Guelph, Guelph, ON Canada; 5 Koret School of Veterinary Medicine, Hebrew University of Jerusalem, Rehovot, Israel; 6 Ecole Nationale Vétérinaire, Nantes, France; 7 Department Microbiology & Immunology, Cornell University, Ithaca, NY USA; 8 Department of Clinical Sciences, North Carolina State University, Raleigh, NC USA; 9 Istituto Zooprofilattico Sperimentale delle Venezie, Legnaro, Italy; 10 Department of Veterinary Sciences and Animal and Veterinary Research Centre (CECAV), University of Trás-os-Montes e Alto Douro (UTAD), Vila Real, Portugal; 11 Aggeu Magalhães Institute, Fundação Oswaldo Cruz (Fiocruz), Recife, Brazil; 12 College of Veterinary Medicine, Murdoch University, Murdoch, WA Australia; 13 Bundeswehr Institute of Microbiology, Munich, Germany; 14 Department Animal Medicine and Surgery, Universitat Autònoma de Barcelona, Bellaterra, Spain; 15 Istituto Superiore di Sanità, Rome, Italy; 16 Institute for Medical Microbiology and Infection Control, Goethe-University, Frankfurt, Germany; 17 Clinic of Small Animals, Freie Universität Berlin, Berlin, Germany; 18 TransMIT GmbH, Giessen, Germany; 19 Department of Clinical Sciences, Colorado State University, Fort Collins, CO USA; 20 Clinglobal, Tamarin, Mauritius; 21 Global Health and Tropical Medicine, Instituto de Higiene e Medicina Tropical, Universidade NOVA de Lisboa, Lisbon, Portugal; 22 Facultad de Veterinaria, Universidad Complutense de Madrid, Madrid, Spain; 23 LABOKLIN GmbH, Bad Kissingen, Germany; 24 Department of Veterinary Medicine and Animal Production, University of Naples Federico II, Naples, Italy; 25 Department of Veterinary Medicine, University of Bari Aldo Moro, Bari, Italy; 26 Department of Veterinary Sciences, University of Messina, Messina, Italy; 27 Institute of Animal Hygiene and Veterinary Public Health, University of Leipzig, Leipzig, Germany; 28 Hospital Clínic Veterinari, Universitat Autònoma de Barcelona, Barcelona, Spain; 29 College of Veterinary Medicine, Chonnam National University, Gwangju, South Korea; 30 Lehrstuhl für Bakteriologie und Mykologie, Ludwig-Maximilians-Universität München, Munich, Germany; 31 Bristol Veterinary School, University of Bristol, Bristol, UK; 32 Melbourne Veterinary School, University of Melbourne, Parkville, VIC Australia; 33 Department of Pathobiology, Oklahoma State University, Stillwater, OK USA

**Keywords:** Relocation, Canine, Importation, Animal welfare, Zoonosis, Parasites, Prevention, Adoption, Shelter

## Abstract

The Companion Vector-Borne Diseases (CVBD) World Forum is a working group of leading international experts who meet annually to evaluate current scientific findings and future trends concerning the distribution, pathogenesis, clinical presentation, diagnosis and prevention of vector-borne infections of dogs and cats. At the 14th Symposium of the CVBD World Forum in Trieste, Italy (March 25–28, 2019), we identified the need to (i) bring attention to the potential spread of parasites and vectors with relocated dogs, and (ii) provide advice to the veterinary profession regarding the importance of surveillance and treatment for parasites and vector-borne infections when rehoming dogs. This letter shares a consensus statement from the CVBD World Forum as well as a summary of the problem faced, including the role of veterinary professionals in parasite surveillance, causal issues, and the importance of interdisciplinary cooperation in addressing the problem. To limit opportunities for dissemination of parasites and vectors, whenever possible, underlying problems creating the need for dog rehoming should be addressed. However, when it is necessary to rehome dogs, this should ideally take place in the country and national region of origin. When geographically distant relocation occurs, veterinary professionals have a vital role to play in public education, vigilance for detection of exotic vectors and infections, and alerting the medical community to the risk(s) for pathogen spread. With appropriate veterinary intervention, dog welfare needs can be met without inadvertently allowing global spread of parasites and their vectors.

## Letter to the Editor

The continuous relocation of dogs both within and between countries represents a global veterinary and public health concern. At the 14th Symposium of the Companion Vector-Borne Diseases (CVBD) World Forum, held in Trieste, Italy, from March 25th to 28th, 2019, it was acknowledged that the veterinary profession faces considerable challenges in dealing with these issues. Specifically, there is confusion among veterinary professionals regarding the role they play in international rehoming advice, surveillance, and control of parasites and pathogens for imported and exported dogs. It was agreed that a consensus statement would be beneficial in clarifying the view of the group and the role of the veterinary profession regarding relocation of dogs and the associated potential spread of pathogens, vectors and diseases. Here, the consensus statement is presented alongside a summary of the problem faced, including the role of veterinary professionals in parasite surveillance, causative issues, and the importance of interdisciplinary cooperation in addressing the problem.

### Consensus statement

Economic, cultural and environmental factors are causing global relocation of domestic dogs, which is associated with the risk of dissemination of parasites, pathogens and vectors. Where possible, the underlying problems should be addressed. However, when it is necessary to rehome dogs, this should ideally take place in the country and national region of origin. Where geographically distant relocation is occurring, veterinary professionals have a vital role to play in public education, vigilance for detection of exotic vectors and infections, and alerting the medical community to the risks for pathogen spread. This includes the implementation of appropriate diagnostic tests and parasite or pathogen preventative measures, ideally before relocation, where necessary.

### Summary of the problem

Multiple drivers affect canine welfare worldwide including natural disasters [1], persecution of street dogs [2], the canine meat trade [3], the practice of acquiring pet dogs as puppies bred in high production, commercial facilities, often in geographically remote locations [4, 5] and travelling dogs brought for mating [6]. Public desire to adopt dogs from abroad that have often had their welfare compromised by these events is increasing. In part, this is driven by social media channels in affluent regions [1], and increased awareness of geographically distant homeless dogs. As a result, dogs are often relocated over large geographical distances [7, 8]. However, dog relocation can cause dissemination of pathogen and vector populations [9]. Increased human migration, climate change and pet travel are other factors that favour this expansion [10–12]. Other risks associated with geographically distant rehoming of dogs include behavioural issues and spread of zoonotic parasitic, viral and bacterial infections, such as *Leishmania* spp. [6, 13], rabies [14], *Brucella* spp. [15] or *Leptospira* spp. [16]. Spread of drug-resistant pathogens is an additional concern, e.g. drug-resistant heartworm (*Dirofilaria immitis*) and hookworms (*Ancylostoma caninum*) in North America [17, 18].

### Addressing causal issues

Ideally, driving factors that lead to dog welfare concerns and increased stray dog populations should be directly addressed. Such an approach has a range of benefits beyond a reduction in dog displacement [19]. The authors acknowledge that in many parts of the world problems are linked to economic factors and cultural attitudes [20]. For example, profit from export of dogs is essential for some communities and, in some cases, meets market demand for rescue dogs in countries where there is incomplete knowledge of the welfare implications of importation. In many countries where canine welfare is compromised, human poverty and suffering can make allocation of resources towards animals less of a priority [20]. Although improving human welfare and infrastructure will help animals indirectly, this process can be slow. Where canine rehoming must occur, dogs should remain in their region of origin whenever possible to reduce pathogen spread but also to keep the dogs in as familiar conditions as possible. Street dogs may experience social stress, for instance, when denied free outdoor access. Some communities keep community dogs that are likely to be less stressed free roaming than in a domestic household, as long as overall welfare *via* interventions such as vaccination, quality of diet and parasite prevention can be improved. The authors acknowledge that addressing underlying issues that affect canine welfare and increasing education regarding these issues are long-term objectives. In the meantime, export of dogs will continue to occur, and veterinary professionals have a vital role to play.

### The role of veterinary professionals

Veterinary professionals have an important role to play in maintaining biosecurity, reducing zoonotic risk to pet owners and the wider public, and improving the health of stray dogs. It is essential that veterinary professionals

(i) educate the public about the risks of adopting dogs from abroad or distant regions within a country and inform them about the benefits of adopting dogs locally. This communication should be compassionate as most charities working in this field, and people adopting pets, do so with the best of intentions but may be unaware of the risks. Social media, practice websites, waiting room leaflets and posters, and discussions can all be utilised to disseminate the message.

(ii) ask about travel history for any recently acquired pet and advise appropriate diagnostic testing and preventative treatments depending on parasites and other pathogens present in the country of origin and relevant clinical signs. Further information can be found at the following websites: https://www.esccap.org; https://capcvet.org; https://www.troccap.com; currently https://cvbd.bayer.com – in the future https://cvbd.elanco.com; https://iscaid.org;

(iii) are vigilant in looking for exotic ticks or other arthropods on imported dogs and clinical signs that may indicate infection with pathogens not known to be locally endemic in the region of origin;

(iv) report all findings of unusual ticks or arthropods and unusual infections to local health authorities, universities, independent organisations such as those mentioned in point (ii) and through peer-reviewed publications. This helps generate an up-to-date picture of where vectors and pathogens may be emerging. Examples of published reports include *Haemaphysalis longicornis* ticks in North America [21], *Rhipicephalus sanguineus* ticks in northern European households [22], *Babesia canis* in the UK [23, 24], heartworm (*D. immitis*) in Colorado [8], *Ehrlichia canis* in Australia [25] and *Leishmania* spp. transmission to untraveled dogs in the absence of sand fly vectors in the Czech Republic [6] and in the UK [13, 26].

### Importance of interdisciplinary and international cooperation

Government legislation regarding dog importation and exportation varies but has an impact by potentially limiting the numbers of imported dogs and ensuring compulsory vaccine and parasite/pathogen preventative treatment requirements are followed before entry. Dog importation requirements can also vary depending on whether the dog is classified as personal or commercial. Personal import usually has fewer requirements, which are why rescue groups sometimes translocate dogs as owned pets [27]. In both cases, if the administration of highly effective preventative measures such as anti-rabies vaccination, tick control, testing for vector-borne agents, or praziquantel treatment for *Echinococcus multilocularis* is adequately followed, biosecurity against specific pathogens can potentially be maintained. For example, modelling has demonstrated that the introduction of *E.* *multilocularis* into countries free of the parasite would be inevitable without the compulsory treatment of dogs that have visited or been imported from endemic countries [28]. Other examples include compulsory anti-rabies vaccination of travelling and imported dogs keeping many countries rabies-free, and the success until recently of screening dogs imported into Australia for *E. canis* in preventing its introduction in that country.

Financial aid for projects associated with canine welfare supported by governments and by international charities is helpful in tackling canine welfare issues in countries of origin [19]. This funding, together with increased policing of existing animal welfare laws, is important for implementation of these measures, but it is critically important to be sensitive to accepted norms in different cultures [3]. Engagement of dog rescue organisations is also beneficial for promotion of rehoming of dogs in their region of origin and, wherever possible, to encourage them to relax their requirements for rehoming where welfare will not be compromised as a result. If rehoming requirements are too stringent, this may deter potential owners from adopting dogs from within their own country. The members of the CVBD World Forum pledge their support to continue to provide data and evidence-based advice on reducing parasites and vector-borne pathogens spread through provision of information on optimal testing, preventative treatments, and increased veterinary and public education.

## Data Availability

Not applicable.
